# Using lexical semantic cues to mitigate interference effects during real-time sentence processing in aphasia

**DOI:** 10.1016/j.jneuroling.2023.101159

**Published:** 2023-08-04

**Authors:** Niloofar Akhavan, Henrike K. Blumenfeld, Lewis Shapiro, Tracy Love

**Affiliations:** a School of Speech, Language, and Hearing Sciences, San Diego State University, San Diego, CA, USA; b Joint Doctoral Program in Language and Communicative Disorders, San Diego State University/UC San Diego, San Diego, CA, USA

**Keywords:** Aphasia, Sentence processing, Lexical activation, Animacy mismatch, Eye-tracking

## Abstract

We examined the auditory sentence processing of neurologically unimpaired listeners and individuals with aphasia on canonical sentence structures in real-time using a visual-world eye-tracking paradigm. The canonical sentence constructions contained multiple noun phrases and an unaccusative verb, the latter of which formed a long-distance dependency link between the unaccusative verb and its single argument (which was base generated in the object position and then displaced to the subject position). To explore the likelihood of similarity-based interference during the real time linking of the verb and the sentence’s subject noun, we manipulated the animacy feature of the noun phrases (matched or mismatched). The study’s objectives were to examine whether (a) reducing the similarity-based interference by mismatching animacy features would modulate the encoding and retrieval dynamics of noun phrases in real-time; and (b) whether individuals with aphasia would demonstrate on time sensitivity to this lexical-semantic cue. Results revealed a significant effect of this manipulation in individuals both with and without aphasia. In other words, the mismatch in the representational features of the noun phrases increased the distinctiveness of the unaccusative verb’s subject target at the time of syntactic retrieval (verb offset) for individuals in both groups. Moreover, individuals with aphasia were shown to be sensitive to the lexical-semantic cue, even though they appeared to process it slower than unimpaired listeners. This study extends to the cue-based retrieval model by providing new insight on the real-time mechanisms underpinning sentence comprehension.

## 1. Introduction

In this paper we describe an experiment that investigates whether semantic cuing (in the form of animacy mismatch across nouns in a sentence) can mitigate sentence processing impairments in individuals with aphasia, and examine the role of “similarity-based encoding interference” and its downstream consequences during dependency formation Before discussing the study, we describe the relevant sentence processing literature based on neurologically unimpaired adults to set the stage for a subsequent description of the relevant literature on aphasia.

### 1.1. Sentence processing in unimpaired adults

Processing language entails integration of sentence constituents and establishing linguistic relations between the auditorily incoming sentence components. In other words, as the sentence unfolds in real-time, the listener creates links between the sentence constituents (e.g., nouns and verb). As part of this ‘dependency linking’, information needs to be encoded when it is first perceived and then retrieved at subsequent points for efficient processing (Gibson et al., 2000; Lewis & Vasishth, 2005). For instance, to illustrate processes involved in dependency linking, consider the below object-extracted constructions (e.g., [a] and [b] below. Here, successful comprehension requires the retrieval of the direct object of the verb (/the general/) to be integrated and linked with the verb when it is encountered (Kluender & Kutas, 1993).

[a] It was *the general*_i_ that the lawyer chased_i_ <> from the office yesterday.
[b] It was *the general*_i_ that Christopher chased_i_ <the general> from the office yesterday.

A model that describes this dependency linking process is the cue-based parsing approach (Lewis & Vasishth, 2005; Van Dyke & Lewis, 2003). According to this model, the linguistic representations of words and phrases in a sentence are encoded as bundles of feature-value pairs. These features are then used as retrieval cues to carry out the cue-based search and retrieve a co-dependent item (i. e., N1 = /the general/) during the integration process once the verb is reached. This series of efficient cue-based retrievals can shape the integration mechanism of sentence processing.

It is important to note that although retrieval using the features of sentence constituents makes the integration mechanism efficient, it makes the process sensitive to interference from elements whose featural specification is similar to the retrieval cues. For instance, take sentence [a] versus [b] above. In sentence [a], the direct-object noun^1^/the general/has a similar syntactic and semantic (feature) representation to the subject of the embedded clause,/the lawyer/; both have the same structural configuration/det N/and share semantic features (i.e., are animate). However, in sentence [b], the features of the direct object differ from the subject of the embedded clause (the proper noun/Christopher/) both structurally and semantically.^2^ The feature similarity amongst elements of the sentence can decrease the distinctiveness of encoded representations which ultimately may reduce the probability of efficient retrieval at the syntactically dependent gap site (Hofmeister & Vasishth, 2014; Oberauer & Lange, 2008). This similarity-based interference between sentence components with featural overlap can hinder semantic integration in real-time and can impact sentence comprehension accuracy in the context of an inefficient monitoring system (Gordon et al., 2006). Previous studies that investigated reading skills in unimpaired individuals (Gordon et al., 2002, 2004, 2006) have demonstrated that a mismatch in the features of encoded constituents of the sentence (such as/the general/and/Christopher/in [b]) can reduce the similarity-based interference effects which can increase the probability of on-time target retrieval (i.e.,/the general/after the verb/chased/). Therefore, comprehension has been found to be more successful for sentences such as [b] compared to sentences such as [a]. In addition, other studies (Akhavan et al., 2022; Hofmeister, 2011; Hofmeister & Vasishth, 2014) have demonstrated that adding a modifier increases the syntactic and semantic representational complexity of the to-be-retrieved item (e.g., “It was/the *victorious four-star* general/_i_ that the lawyer chased_i_ …”) and facilitate its representational activation and increase its chances of retrieval when the verb is encountered.

According to studies investigating the cue-based model and similarity-based interference effect, unimpaired individuals can use lexical representational information (e.g., semantic) in real-time to aid their sentence processing as these cues determine the bundles of features that are used for successful dependency linking (Lewis & Vasishth, 2005; Van Dyke & Lewis, 2003). Given the evidence that lexical cues (such as animacy) play an important role in unimpaired sentence processing, one useful avenue in understanding sentence comprehension deficits in individuals with aphasia (denoted as IWA throughout) is to investigate whether and when IWA use these types of cues during their integration process in real-time to resolve interference effects. Below, we review the literature examining sentence processing in aphasia and how lexical-semantic cues are processed during sentence comprehension.

### 1.2. Sentence processing in aphasia

Several studies have provided evidence indicating that IWA experience sentence comprehension problems. These problems can be characterized as *processing* accounts, stating that there is no loss of knowledge in IWA, but that the ability to apply the knowledge is impaired (Choy & Thompson, 2010; Ferrill et al., 2012; Hagoort et al., 1996; Love et al., 2008; Swaab et al., 1997, 1998). According to this class of theory, IWA are aware of the cue, but they are not able to process it accurately and/or efficiently. The specific nature of the deficit in sentence comprehension in IWA remains controversial due to individual differences among affected individuals, as well as the likelihood of multiple relevant processes or deficits contributing to the impairment.

Within the processing accounts, some argue that sentence comprehension impairments are caused by deficits in real-time lexical processes which are assumed to act as the interface between sound input and the construction of a grammatical and interpretative representation of an utterance. Specifically, some suggest that IWA have delayed lexical access (Love et al., 2008) and/or lexical integration (Thompson & Choy, 2009), which results in disruption of the on-time availability of lexical representations for syntactic processing and can ultimately bring about comprehension failure. These hypotheses explain that IWA can experience a delay in their ability to access and retrieve lexical information. When lexical processing is delayed, IWA may not be able to integrate the relevant information as they are processing the sentence, leading to difficulties in understanding the relationship between sentence constituents. As a result, they may experience difficulty in accurately processing complex sentence structures, such as those involving embedded clauses or long-distance dependencies that require on time gap-filling processes.

Several factors may be at the root of these lexical processing impairments. First, limitations of working memory capacity have been identified post-stroke, such that IWA may have difficulty maintaining the activation of lexical representations which can impede sentence processing (Martin et al., 1999; Martin & Gupta, 2004). In the context of the cue-based model, limitations of working memory can be associated with difficulty in integrating and using multiple cues in real time. Others suggest that IWA may have trouble selecting among competing activated representations during the lexical access and integration stages of sentence processing due to executive function deficits because of damage to a specific language network (e.g., the left inferior frontal gyrus which is commonly implicated in IWA impairments) (Gotts & Plaut, 2002; Jefferies et al., 2008; Jefferies & Lambon Ralph, 2006; Novick et al., 2005; Sheppard et al., 2015). Within the framework of the cue-based model, limitations in executive functions may hinder the selection of relevant cues for retrieval. Altogether, given challenges in lexical processing and sentence comprehension, once the similarity of features between noun phrases is high, IWA are likely to be susceptible to experiencing an interference effect. To explore this susceptibility, Sheppard et al. (2015) conducted an investigation with IWA to examine their auditory comprehension of various types of wh-questions (see footnote^3^ for sentence examples) using a visual-world eye-tracking paradigm. During this task, participants were required to identify the correct referent of the questions. The study found that IWA exhibited significantly poorer performance in terms of their comprehension accuracy, reaction time, and gaze location when processing object-extracted which-questions, as compared to other types of wh-questions. This stems from the fact that analyzing the dependency relationship between the moved noun phrase and its corresponding gap requires encountering another argument - namely, the *intervener* - which must be considered when computing the relationship. In other words, the use of “which” introduced an additional level of complexity, as it requires the processing of a relative clause that modifies “mailman”. This sentence type (e.g., “Which mailman did the fireman push ___ yesterday afternoon?”) involves computing the dependency relationship between the displaced noun phrase “which mailman” and its gap “mailman” in the sentence. This process requires crossing over another argument, the “fireman”, which is referred to as an “intervener”. This additional processing step created a processing disadvantage for IWA, as compared to “Who did the fireman push yesterday afternoon?” which only requires the processing of a single noun phrase. It is important to note that the intervener hypothesis introduced by Sheppard et al. (2015) is based on Rizzi’s relativized minimality account (1990), which has been further supported by subsequent studies such as Grillo (2005, 2009).

While unimpaired listeners also show an interference effect, it does not hamper real time processing or final interpretation. Further, in unimpaired individuals, providing semantic cues has been shown to reduce similarity-based interference and aid comprehension (Akhavan et al., 2022; Hofmeister, 2011; Hofmeister & Vasishth, 2014). Yet, while semantic cueing is well-established as a productive offline strategy in clinical intervention with IWA (Python et al., 2021), it is still unknown whether IWA can use a salient lexical-semantic cue in real-time to mitigate any interference effects during sentence comprehension. Here, in the context of cue-based parsing, “semantic cueing” refers to the use of semantic information to facilitate the processing of a sentence. In this study, we utilized animacy mismatch between sentence constituents to guide the listener’s processing of the sentence and disambiguate potential sources of interference.

### 1.3. Processing lexical cues during sentence comprehension in aphasia

Previous research suggests that, in the context of sentence comprehension impairment, IWA may rely on semantic information in their processing of sentences that require dependency linking. A seminal study examining the sensitivity of individuals with aphasia to semantic information during sentence comprehension was conducted by Caramazza and Zurif (Caramazza & Zurif, 1976). In this study, individuals with Broca’s aphasia were presented with sentences like the following in a sentence-picture matching task:

[c] The book that the girl is reading is yellow.
[d] The cat that the dog is biting is black.

To understand non-canonical sentences like [c] and [d], the listener must integrate the lexical items as they are heard based on structural rules to determine who is doing what to whom. However, research has shown that listeners can enlist other strategies to help make the comprehension of these non-canonical sentence constructions easier (Bhandari et al., 2021). The Caramazza and Zurif (1976) study revealed that IWA had little difficulty understanding non-canonical sentences when the information provided from the nouns allowed for an easy determination of who was performing the action and who was receiving the action. For example, in [c], the listener can take advantage of the fact that in this non-reversible sentence, only the animate noun (/the girl/) can perform the action of reading. However, in semantically reversible (non-canonical) sentences like [d], where both noun phrases (/the cat/and/the dog/) can perform the action of biting, individuals with Broca’s aphasia demonstrated comprehension difficulty. As discussed earlier, both noun phrases (/the dog/and/the cat/) have similar lexical features that prevent reliance on featural cues such as animacy, as shown in [c]. This work demonstrated that IWA, in some linguistic contexts, could rely on semantic cues to compensate for difficulty in syntactic dependency linking.

In another sentence comprehension study, Gibson and colleagues (Gibson et al., 2016) examined the sensitivity of individuals with aphasia to semantic plausibility by using an act-out task (where comprehension is measured by asking participants to act out sentences with dolls) using active/passive^4^ sentences as well as Double-Object (DO)/Prepositional-phrase Object (PO)^5^ sentence structures (see footnotes 4 and 5 for examples of each sentence structure). They found that compared to the control group, individuals with aphasia relied more heavily on plausibility information (i.e., all sentences follow the common pattern of relationship between entities in the sentences) across all different sentence types. Specifically, IWA were more likely to use plausibility information in non-canonical passive relative to canonical active constructions.

Altogether, these studies revealed that IWA showed sensitivity to semantic information during their processing of sentences that require dependency linking. However, these studies did not capture the moment-by-moment processing patterns and we still do not know the immediate effect of these cues for IWA. Here, we examine whether and when IWA can use lexical-semantic cues to resolve any potential interference effects stemming from syntactic properties of the sentence.

### 1.4. Current study

In the current study, we employed an eye-tracking while listening visual world paradigm (ETL) to study how lexical-semantic cues (in the form of animacy) impact sentence processing in individuals with aphasia. Eye-tracking while listening is a method that allows us to investigate online sentence processing with millisecond-level temporal resolution. Additionally, this experiment used a natural speech paradigm without any behavioral response required during sentence processing. Being able to index participant responses without requiring overt participant decisions is a significant advantage of employing eye-tracking methods in IWA.

We seek to understand how IWA process lexical-semantic cues (animacy features) during the processing of canonical sentences that require dependency linking by comparing their performance in conditions with [see sentence e, below] and without [see sentence f, below] lexical-semantic cues. We use sentences containing unaccusative verbs as they can provide a case of long syntactic dependency in a canonical-order structure (Bever & Sanz, 1997; Burkhardt et al., 2003; Friedmann et al., 2008; Koring et al., 2012; Poirier et al., 2012; Sullivan et al., 2017a; Sullivan et al., 2017b). The subject and the direct object of the first verb (/noticed/), also known in linguistic terminology as arguments, are/the model/as the agent and/the dress or designer/as the theme respectively. Critically, the argument structure of the second verb (the unaccusative verb/fell/) is different, as it can be considered as something that happened to the subject of the sentence (/the model/), rather than being initiated by it. In other words, the canonically assigned subject position also has the properties of the argument that are syntactically associated with the object/theme (Burzio, 1981, 1986; Perlmutter, 1978).

**Table:** 

Condition	Example sentences
[e] Inanimate	This evening at the fashion show, the model_i_^animate, subject^ that noticed the dress ^inanimate, object^ surprisingly **fell**_i_^[animate, subject]^ during the evening gown showcase.
[f] Animate	This evening at the fashion show, the model_i_^animate, subject^ that noticed the designer ^animate, object^ surprisingly **fell**_i_^[animate, subject]^ during the evening gown showcase.

In addition to the IWA participants, a group of age-matched unimpaired individuals (AMC) were also included in the study to establish a baseline processing performance, in comparison to IWA, on these sentences using eye-tracking while listening. In this mixed-subject design study, we examine not only the potential differences between the two participant groups but also assess individual differences across the members of each group in each condition.

### 1.5. Questions and predictions of the current study

We asked the following three questions:

#### Question 1:

Are listeners of each group sensitive to lexical-semantic cues (animacy) that resolve semantic interference during sentence comprehension? For this question, we compared the performance of each group across the two experimental conditions: (a) where both noun phrases were animate and (b) where one noun phrase was animate while the other was inanimate. We predicted that listeners in both groups would be sensitive to lexical semantic stimulus properties during sentence comprehension. In the inanimate condition, upon encountering the inanimate noun (N2), both groups (IWA and AMC) were predicted to show a reduced similarity-based interference effect. In this eye-tracking while listening paradigm, the susceptibility to the interference effect was operationalized as the degree to which participants distribute their gaze between two pictures, with higher interference indicated by a more equal proportion of gazes to the target noun (N1) and non-target or intervening noun (N2) (Akhavan et al., 2020).

#### Question 2:

Do IWA demonstrate their sensitivity to lexical-semantic cues in real-time or is there a delay? For this question we compared the performance of IWA versus AMC in the inanimate condition to examine whether IWA process the lexical-semantic cue at the same time as AMC. Based on previous studies that revealed the temporal delay of IWA in processing lexical-semantic representations (Engel et al., 2018; Ferrill et al., 2012; Love et al., 2008; Prather et al., 1997), we expected IWA to show a delay compared to AMC individuals in using the lexical-semantic cue (animacy) in real-time.

#### Question 3:

Does the lexical-semantic cue have a downstream effect on syntactic dependency linking at the gap-site? Recall that the sentences in this experiment include a gap-site when the unaccusative verb is encountered. For this question, we examined the effect of condition for each group. We predicted that the lexical-semantic cue would have a downstream effect for both groups as the inanimate N2 (‘the dress’ versus ‘the designer’) would induce a smaller interference effect during the syntactic dependency linking process. In other words, the inanimate noun would improve the real-time processing as well as final comprehension for listeners.

## 2. Method

### 2.1. Participants

#### Age-Matched Control Participants (AMC):

Eleven AMC participants were involved in this study. Their mean age at the time of testing was 62 years old [range: 57–66 years], with an average 15.2 years of education [range: 12–20 years]. All AMC participants were monolingual native English speakers with self-reported visual and auditory acuities that were normal or corrected-to-normal. None of the participants had a self-reported history of substance abuse, psychiatric illness, intellectual disability, or other significant brain disorder (e.g., Alzheimer’s/dementia).

#### Individuals with Aphasia (IWA):

Eleven individuals, diagnosed with Aphasia by a certified speech-language pathologist, were included in this study. Their mean age at the time of testing was 64 years old [range: 56–77 years], with an average 15.7 years of education [range: 14–18 years]. IWA did not significantly differ from AMC participants in terms of age (p > .05), see Table 1 for demographic information. All IWA were right-handed before their stroke, native English speakers, with normal or corrected-to-normal visual and hearing. They all had a single, unilateral left-hemisphere stroke. Clinical consensus of an aphasia diagnosis (including severity and specific impairment in fluency and auditory comprehension) was made for each participant using standardized language testing. The standardized testing tools included the Boston Diagnostic Aphasia Examination—Third Edition [BDAE– 3] (Goodglass et al., 2001), the Western Aphasia Battery-Revised [WAB-R] (Kertesz, 2007), and a test of auditory sentence comprehension [SOAP: Subject-relative Object-relative Active and Passive] (Love & Oster, 2002). All individuals with aphasia were neurologically and physically stable (i.e., at least 6 months post-stroke) and, similar to the AMC group, had no self-reported history of substance abuse, psychiatric illness, intellectual disability, or other significant brain disorder (e.g., Alzheimer’s/dementia).

This research was approved by the IRB committees at both San Diego State University and University of California San Diego. All data were collected at the Language and Neuroscience Group Laboratory located at San Diego State University. Participants received $60 ($15 per session) for their participation in the study.

### 2.2. Materials and design

We used canonical^6^ subject-relative sentence structures that are embedded into an intransitive main clause. Note that canonical here is defined in terms of grammatical roles, with any S–V structure considered canonical (see footnote 6). The sentences were structured to yield two conditions: a mismatch condition with animate and inanimate nouns [e] and a match condition with two animate nouns [f]. We used 15 intransitive (unaccusative) verbs. These verbs were used twice in each of the two conditions as indicated below [e, f], resulting in 60 sets of sentences (see Appendix for the full list of stimuli). As in the study by Sullivan et al. (2017a), the selection of verbs for the embedded clauses of the experiment was limited to non-alternating unaccusative verbs which do not have a transitive alternation. The selection process was based on the verbs’ linguistic characteristics, including their behavior with respect to specific diagnostic tests (as outlined in Friedmann et al., 2008). These diagnostic tests included (1) the verb’s occurrence in there-constructions, (2) ungrammaticality when used with a direct object, and (3) the inability to undergo passivization; see the footnote for examples.^7^

[e] Inanimate (mismatch) condition: This evening at the fashion show, the model ^animate, subject^ that noticed the dress ^inanimate, object^ surprisingly **fell** ^[animate, subject]^ during the evening gown showcase.
[f] Animate (match) condition: This evening at the fashion show, the model ^animate, subject^ that noticed the designer ^animate, object^ surprisingly **fell** ^[animate, subject]^ during the evening gown showcase.

An additional 120 filler sentences were included (though not analyzed as they did not contribute to the theories under investigation). These filler sentences differed from the experimental and control condition sentences in terms of grammatical structure. Thus, participants were presented with a total of 180 sentences. To prevent any potential order effects, the experimental sets were distributed evenly across four presentation lists, and the location of images was counterbalanced for each item. Additionally, the order of presentation was randomized for each participant and balanced, with an average of five days between sessions.

**Fig. 1.**
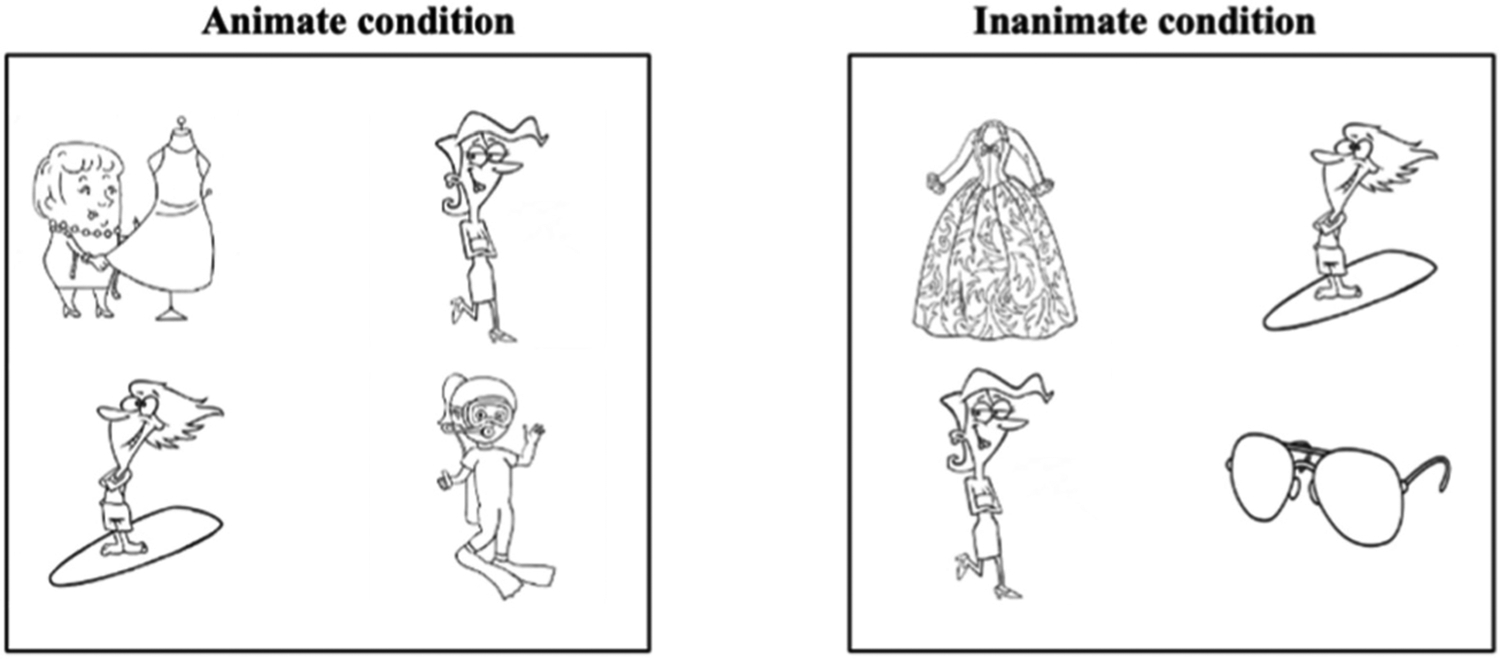
Example of the animate (1a. left column) and inanimate conditions (1b. right column) in the visual world paradigm where participants view four images, hear a sentence, and identify the corresponding target noun from the visual display. Sample stimuli are presented along the trial timeline, with corresponding auditorily presented sentences listed below.

A native English-speaking female recorded all sentences with an average speech rate of 4.47 syllables per second. Line drawings of each of the nouns of interest were created for each sentence trial (see Fig. 1a and b). All images were sized at 450 × 450 pixels.

In the current experiment, we used a switch target design. This allowed us to use the nouns [Ns] as either target or the distractor items. For example, in the animate condition, the display contained the two animate nouns mentioned in the sentence (e.g., model and designer), and the two animate referents from another experimental sentence (e.g., surfer and scuba diver). This design ensured that gaze interest to Ns were because of lexical processing, and not due to a visual preference for a particular image. Additionally, the location of the images on the display was counterbalanced across trials. The sentence trials were counterbalanced across four presentation lists. The order of presentation across participants was counterbalanced within four sessions. Therefore, participants came into the lab for four sessions with an interval of a week between sessions to complete this within-subjects experiment.

### 2.3. Stimulus pre-testing

We conducted two sets of pre-tests to check if the stimuli were appropriate for use. The first pre-test was a picture naming task. A group of college-age unimpaired participants (N = 20) were asked to name the images of the noun phrases to ensure that the images clearly depicted the N of interest (i.e., that the image of/the model can be accurately identified as intended). For this study, we used the images that had a minimum of 75% agreement in exact naming and semantically related naming matches. The second pre-test was conducted with another group of college-aged unimpaired students (N = 27). The purpose of this pre-test was to reduce plausibility effects by confirming that Ns mentioned in a sentence (e.g.,/the model/and/the designer/) were equally likely to be at the place mentioned in the initial PP of each sentence (e.g.,/This evening at the fashion show/). Participants were asked to use a 1–5 Likert scale (1-not very likely, 5-extremely likely) to rate how likely it was that the person (e.g., designer) would be at the place (e.g., fashion show). The items that received ratings of 4 and 5 on likelihood of being at the place tested (N1 *M* = 4.8, *SD* = 0.6); (N2 *M* = 4.7, *SD* =0.8) were included in this study.

### 2.4. Procedure

Using headphones, participants were presented with uninterrupted auditory sentences while looking at a four-picture display of line drawings in black and white. Participants sat in front of a computer screen and a Tobii X-120 eye-tracker with a 60 cm distance between their eyes and the eye-tracker and the screen used to show stimuli. The gaze sampling rate of Tobii X-120 eye-tracker was 60 Hz meaning that the gaze data was recorded every 17ms across each trial. E-prime 2.0 software (Psychology Software Tools, Pittsburgh, PA) was used to present stimulus trials.

**Fig. 2.**
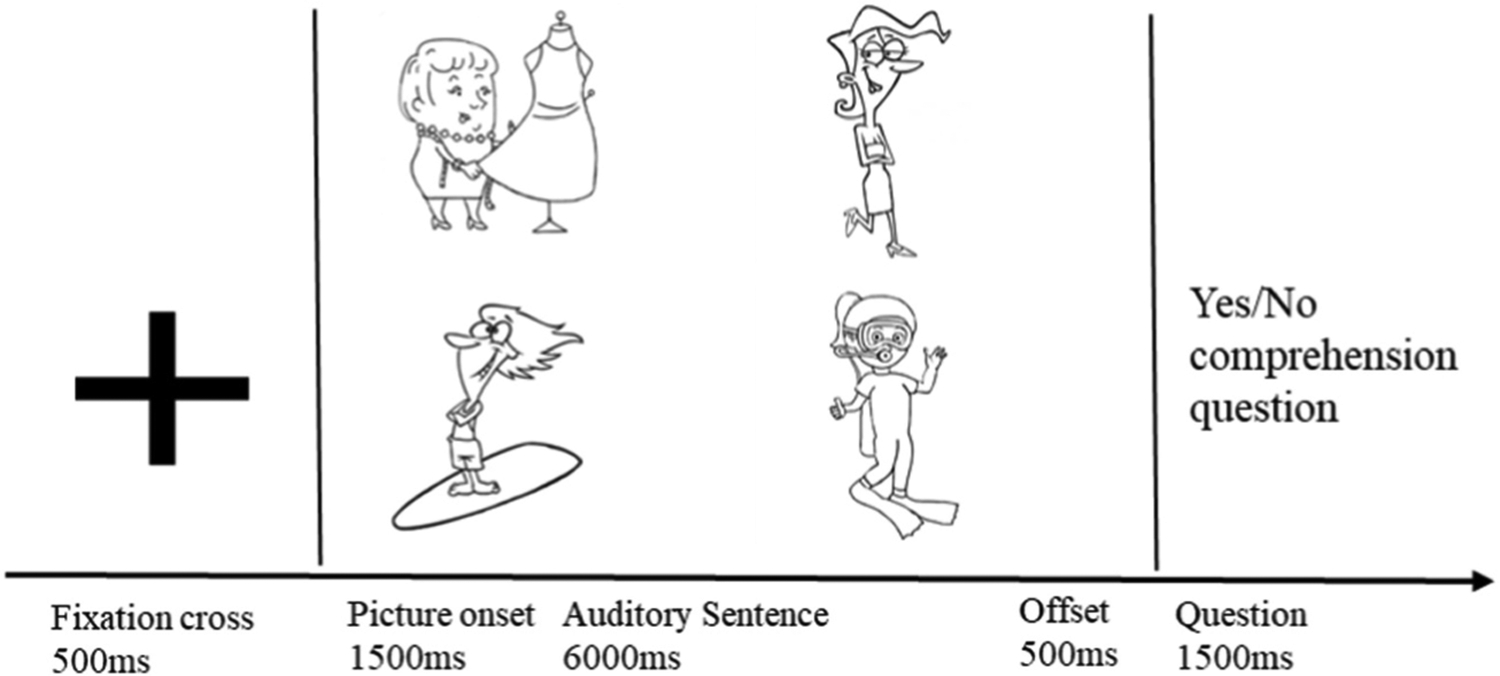
Schematic illustration of a trial including the online and offline sections in a visual world eye-tracking paradigm.

At the start of each experimental session, the eye-tracker was calibrated. Each trial started with a 500ms fixation cross, followed by a 250ms blank screen. Then the four-picture display was presented for 1500ms before the corresponding auditory sentence was presented. The four-picture display remained on the screen 500ms after the sentence ended. After the gaze data was sampled in each trial, participants were asked a Yes/No question after each sentence (for example: Did the model fall during the evening gown showcase?). The intent of these questions was to reinforce the participants to attend to the sentences (see Appendix). These comprehension sentences were constructed in a manner to not specifically focus on the subject-verb dependency. That is, for some questions N1 was treated as the agent and for others N2 was treated as the agent. The responses of participants were recorded via a button box (with Yes/No keys) using their left hand (non-dominant). See Fig. 2 for schematic illustration of the eye-tracking paradigm. Prior to each experimental session, each participant was presented with 10 practice trials, and this provided the opportunity to provide feedback or redirection to participants if necessary.

### 2.5. Data analysis approach

In this section we describe the approach taken to post-process and analyze both the eye-tracking (gaze position captured every 17 ms) and end of sentence comprehension question (Yes/No) data.

#### Comprehension question data preparation.

After listening to each sentence, participants were asked a Yes/No comprehension question. Both button press (Yes or No) and time to respond (in ms) were captured. Responses that had less than 100ms response latencies were excluded from the analysis as this is not a feasible voluntary response. Regardless of comprehension performance, all sentence trials were entered into the model for further processing and analysis.

#### Eye-tracking gaze data preparation.

Processing of the eye-tracking data is necessary to (1) check for trackloss and (2) aggregate the gaze data points across temporal bins.

1. Trackloss is an instance when the gaze data are not accessible from both participant’s eyes. This occurs when participant’s eyes turn away or blink. The Tobii system provides a metric for validity of each gaze datapoint which can be used to account for trackloss in the analysis. As a result of trackloss analysis per trial, trials with greater than 25% of trackloss were excluded from further analyses. Moreover, the total percent of trackloss was calculated for each participant in both groups. AMC participants had an average trackloss of 10% while IWA had an average trackloss of 7%, which was not statistically different (*p* > .05).
2. The proportion of gazes were computed by dividing the total duration of gazes on a specific Area of Interest (AOI, numerator) by the total duration of the trial (denominator). Gaze proportions toward each image on the screen for each trial were aggregated into time bins of 100ms. For each bin, the proportion of gazes was estimated within each AOI on the display (Mirman, 2017). Gaze data were then subjected to statistical analysis as described below. This method is employed to take into account the underlying dependency between datapoints (i.e., autocorrelation) in time-series eye-tracking data which can increase Type I error rates.

### 2.6. Time windows of analysis

Although sentences had similar lengths and syllable numbers, to account for slight timing differences, we coded the timing onset for each of the sentence constituents (initial prepositional phrase, noun phrase 1 [NP1], relativizer, noun phrase 2 [NP2], adverb, verb and final prepositional phrase; see Table 2) across all experimental items. This allowed us to normalize the onset as we identified the windows of interest across sentences. We identified two main windows of analysis. The first window captured the average onset of NP1 until the average offset of NP2. To allow for potential delays during processing by IWA, this time window has been extended even further. The second window captured the average onset of the adverb until the average offset of the final PP to ensure the inclusion of the gap-site. See Table 2 for the average timings at which these windows happen across sentences of each condition. To allow for potential delays when IWA would process the embedded clause, the end of the embedded clause time window (4800ms, see Table 2) overlapped with the onset of the dependency processing time window (4400ms).

**Table:** 

This evening at the fashion show,	the model	that noticed	the designer	surprisingly	fell	during the evening gown showcase
This evening at the fashion show,	the model	that noticed	the dress	surprisingly	fell	during the evening gown showcase
**INITIAL PP**	**NP1**	**REL**	**NP2**	**ADV**	**VERB**	**FINAL PP**

### 2.7. Growth curve analysis

We used Growth Curve Analysis (GCA) to examine the patterns of gaze proportion trajectory over the course of time in two pre-selected time windows of interest within the sentence (see Table 2, gray and yellow boxes). As the current timecourse analyses were aimed at uncovering realtime processing regardless of final deliberate interpretation of the sentence, trials associated with both accurate and inaccurate comprehension question responses were included in analyses. The GCA approach has been widely used in the visual-world eye-tracking studies to examine the gaze trajectory over time (Akhavan et al., 2020; Baker & Love, 2021; Brown et al., 2011; Hadar et al., 2016; Mirman et al., 2008, 2011). GCA is a multi-level modeling technique which employs fitting an orthogonal polynomial curves to the time-course data to capture gaze proportion change over time (Mirman et al., 2008). By adding the effects of the variables of interest (e.g., experimental conditions or group effects included here as dummy treatment/variables) on the orthogonal polynomial terms, we can quantify the extent of their effects on the gaze proportion trajectory. Here we captured the overall gaze trajectory at the intercept, linear and quadratic polynomial terms. Each of these time terms reflect a specific aspect of gaze behavior (Mirman et al., 2008). As described by Mirman et al. (2008), the intercept term reflects the average overall gaze proportion; the linear term reflects a monotonic change in gaze proportion which is similar to a linear regression of gaze proportion as a function of time; the quadratic term reflects the symmetric rise and fall rate around a central inflection point. Moreover, GCA modeling involves building a random effect structure. Here we included the random effects of participants and items on intercept, linear, and quadratic time terms. Additionally, we added the random slopes for condition per subject to achieve a maximal random effects structure (Barr et al., 2013). To extract the summary table (coefficient estimates and p-values) for each GCA models, we used the package LmerTest (Bates et al., 2014). We conducted all data processing statistical analyses on the statistical software R-3.2.1.

## 3. Results and discussion

### 3.1. Offline comprehension performance

Recall that after each trial, participants were asked a Yes/No question. For the question accuracy, the mixed-effects logistic regression model revealed an effect of condition in the AMC group showing, as expected, that they had significantly better performance in the inanimate (98% accuracy) than the animate (94% accuracy) condition (Estimate = 1.02, SE = 0.45, p < .05). However, while IWA’s means across conditions followed the same pattern of better performance in the inanimate (75% accuracy) condition (as compared to 69% accuracy for the animate condition), the same statistical model remained insignificant when tested for the IWA group (Estimate = 0.26, SE = 0.26, p = .32, see Table 3). This is likely due to the increased variability in performance for IWA across both conditions.

### 3.2. Real-time gaze analysis

**Fig. 3.**
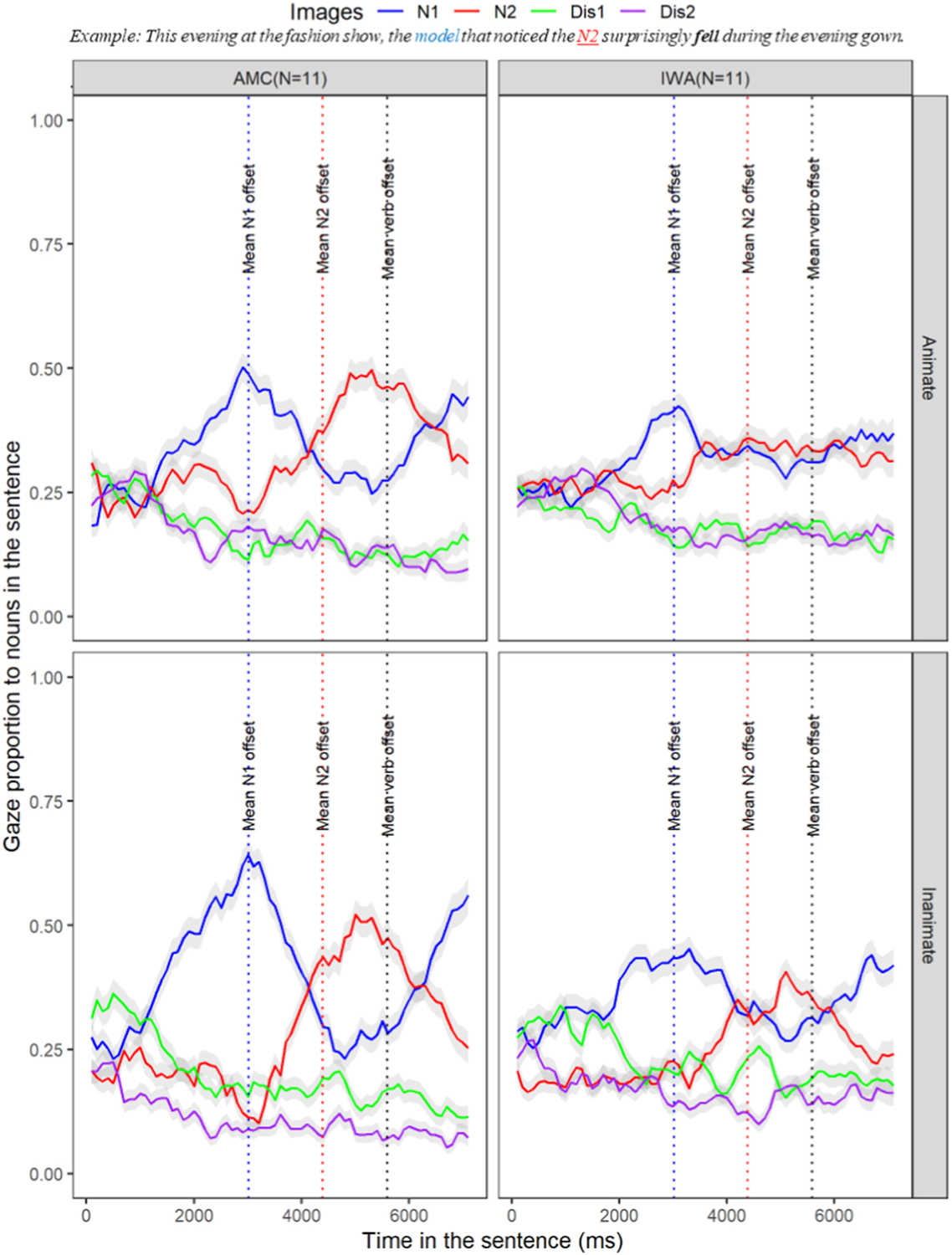
Time course of gaze patterns for all four AOIs across the whole sentence for AMC (n = 11) and IWA (n = 11) in the animate condition (top) and inanimate condition (bottom). Both groups show gaze proportions to the distractor items at the chance level 0.25.

The subsequent analyses were completed on all trials, including those with correct and incorrect responses for offline comprehension questions; for a similar approach, see (Akhavan et al., 2022; Baker & Love, 2021). Analyses were focused on the condition differences between each group at specified windows of interest that were illustrated in Table 2. We first present a birds-eye view of the full time-course of the sentence in Fig. 3; that is, gazes to each of the four AOIs throughout the time-course of each sentence in the animate and inanimate conditions. This time-course includes the initial activation of N1 in real-time (blue line, e.g.,/the model), followed by the deactivation of N1 and activation of N2 (red line,/the dress/or/the designer/), and finally the deactivation of N2 and expected re-activation of N1 at the gap site once the unaccusative verb was encountered (/fell/).

To establish that participants’ gaze was primarily directed towards the items mentioned in the sentence, we measured the proportion of gazes towards all items displayed on the screen, including N1, N2, Dis1, and Dis2. By examining the gaze proportions towards these items, we were able to assess the extent to which the participants’ attention was focused on the relevant items during sentence processing. We found that neither AMC nor IWA participants showed gaze interests to the two distractor items more than at chance level, which is 0.25 in this case (Fig. 3, green line [Dis1] and purple line [Dis2]). As a result, we excluded gazes to distractor items from further analysis and focused solely on the gaze pattern over N1 and N2. In other words, statistical analyses were focused on investigating the group differences (IWA versus AMC) on the proportion of gazes toward N1 and N2. To investigate the group difference on proportion of gazes to N1 and N2, we ran a linear mixed effects model that included the group factor as the fixed effect and subjects and items as the random effects. The model confirmed a significant difference between the IWA and AMC such that the overall proportion of gazes to both N1 and N2 in IWA was revealed to be lower than AMC (Estimate = −0.03, p < .05). This finding reveals expected group differences in lexical activation for IWA relative to AMC. Given the identified differences between the IWA and AMC in online and offline processing, we built separate multilevel models for each group to examine the sensitivity of listeners to semantic cues upon initial encounter (question 1) and downstream in the sentence (question 3). Further, given the overall lexical activation differences in IWA compared to the AMC group, to confirm that individuals with aphasia were experiencing a delay in processing the semantic cue (question 2), we compared their processing to the unimpaired group which served as the baseline. The findings for each question are presented below:

### 3.3. Result of question 1: sensitivity to lexical-semantic cues

**Fig. 4.**
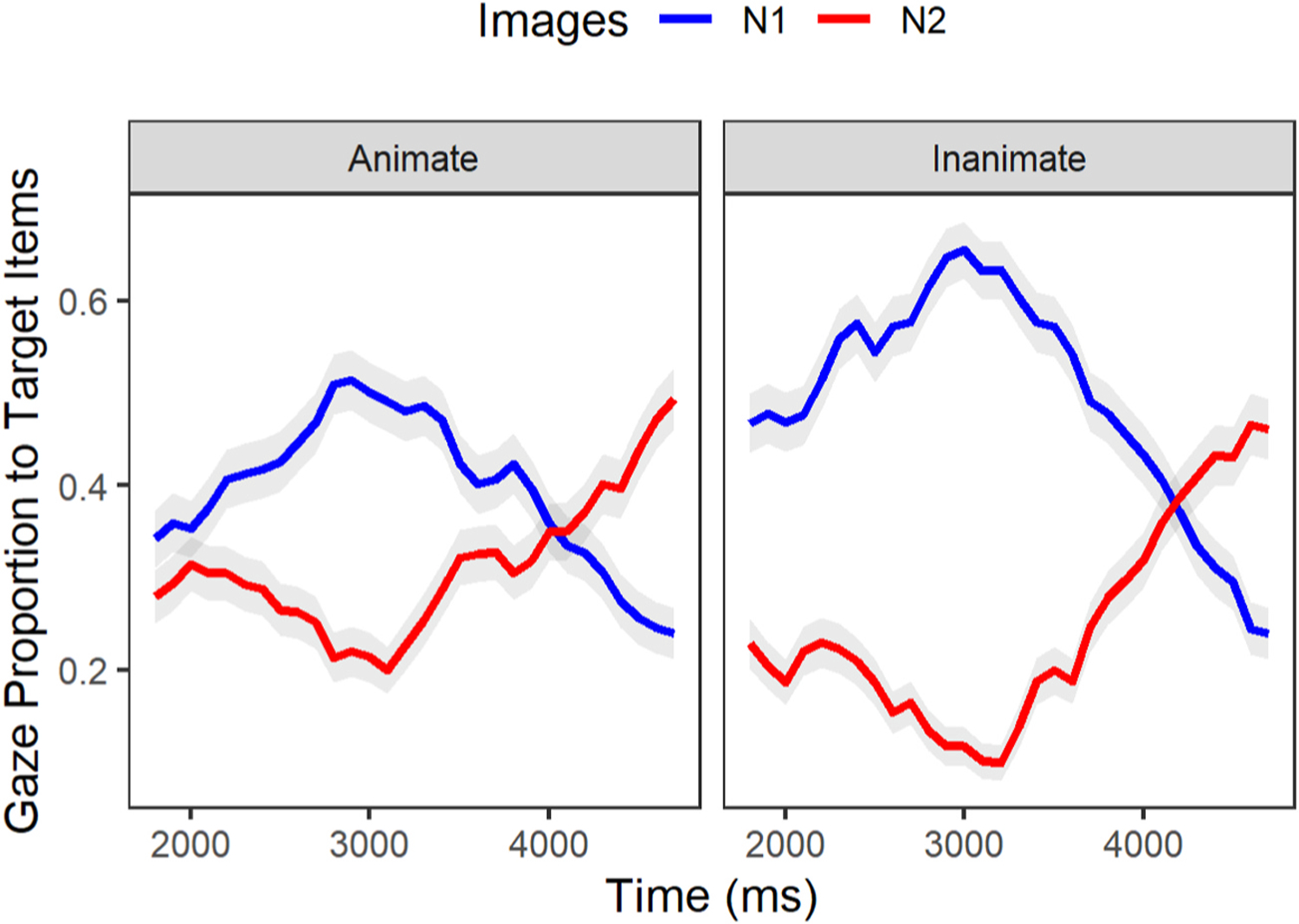
Gaze proportion differences between the animate and inanimate conditions within the AMC group [**…** the model that noticed the designer …].

We examined whether participants in each group demonstrated sensitivity to the lexical-semantic cue of animacy. We proposed that sensitivity to the lexical-semantic cue would yield a smaller interference effect in the inanimate condition where the noun-phrases are mismatching in animacy. In the eye-tracking paradigm, the interference effect is defined as an equivalent proportion of gazes toward related (N1) as well as intervening items (N2). We specified the window of analysis to occur at 200ms before the mean onset of the first N until 3000ms afterward (corresponding to the average offset of N2–see Table 2). Gaze data by group (AMC, IWA) and condition (animate, inanimate) for processing N1 (target noun) and N2 (intervening noun) are plotted in Fig. 4. Of interest for this analysis is the average proportion of looks to N2 relative to N1 between conditions for each group. A significant interaction of images (N1, N2) and condition (animate, inanimate) at the intercept level with a negative estimate would correspond to a smaller proportion of gazes toward N2 (i.e., a smaller interference effect) in the inanimate condition. Moreover, a significant interaction of the same parameters at the linear level^8^ with a positive estimate would correspond to a faster rate of N2 activation in the inanimate condition. In other words, given this context when the linear estimate is positive and statistically significant, this suggests that the proportion of looks toward N2 is increasing over time while the proportion of looks toward N1 is decreasing.

#### AMC group.

We first modeled gaze patterns for the AMC group. The linear mixed effect analysis revealed a significant effect of condition on gaze differences between the N1 and N2 for this group. These results (see Table 4) revealed that, as expected, the inanimate (mismatch) condition yielded reduced interference between N1 and N2 (Intercept: Estimate = −0.16, p < .001; Linear: Estimate = 0.25, p < .001). This effect is apparent in Fig. 4 in the steeper slopes of N1 deactivation and N2 activation as well as the greater separation between N1 and N2 curves for the inanimate condition than the animate condition.

#### IWA group.

**Fig. 5.**
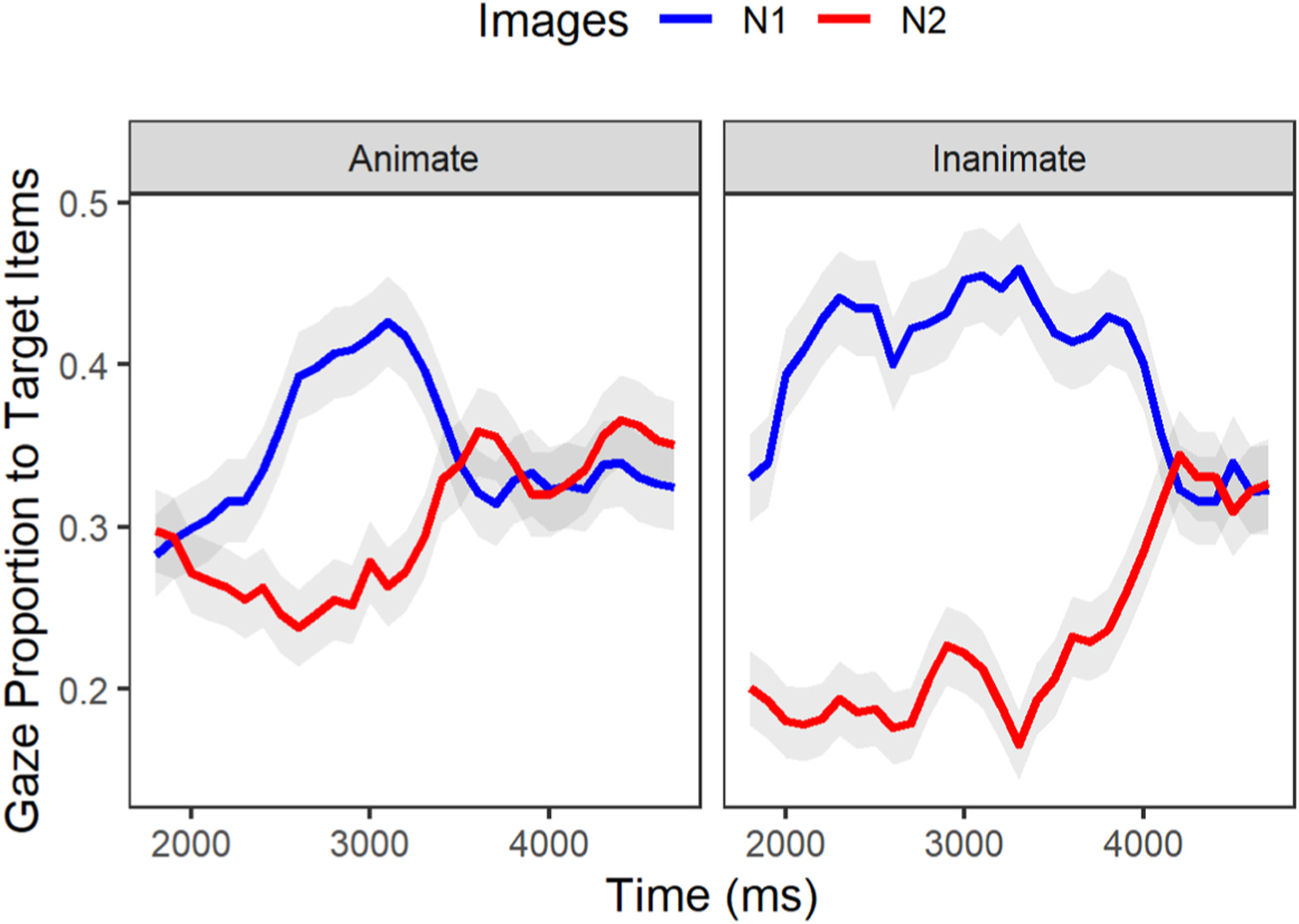
Gaze proportion differences between the animate and inanimate conditions within the IWA [… the model that noticed the designer/ dress …].

The same analysis approach was used for the IWA group, revealing highly similar results (see Table 5). Individuals with aphasia experienced a smaller interference effect in the inanimate condition, which is apparent in the significant interaction between condition (animate, inanimate) and the sentence constituent (N1, N2; Intercept: Estimate = −0.12, p < .001; Linear: Estimate = 0.22, p < .001). As apparent in Fig. 5, IWA also showed greater separation between the N1 and N2 curves in the inanimate than the animate condition. The results from both groups demonstrate the sensitivity of participants to the lexical-semantic manipulation.

### 3.4. Result of question 2 – time-course of sensitivity to the lexical-semantic cue

**Fig. 6.**
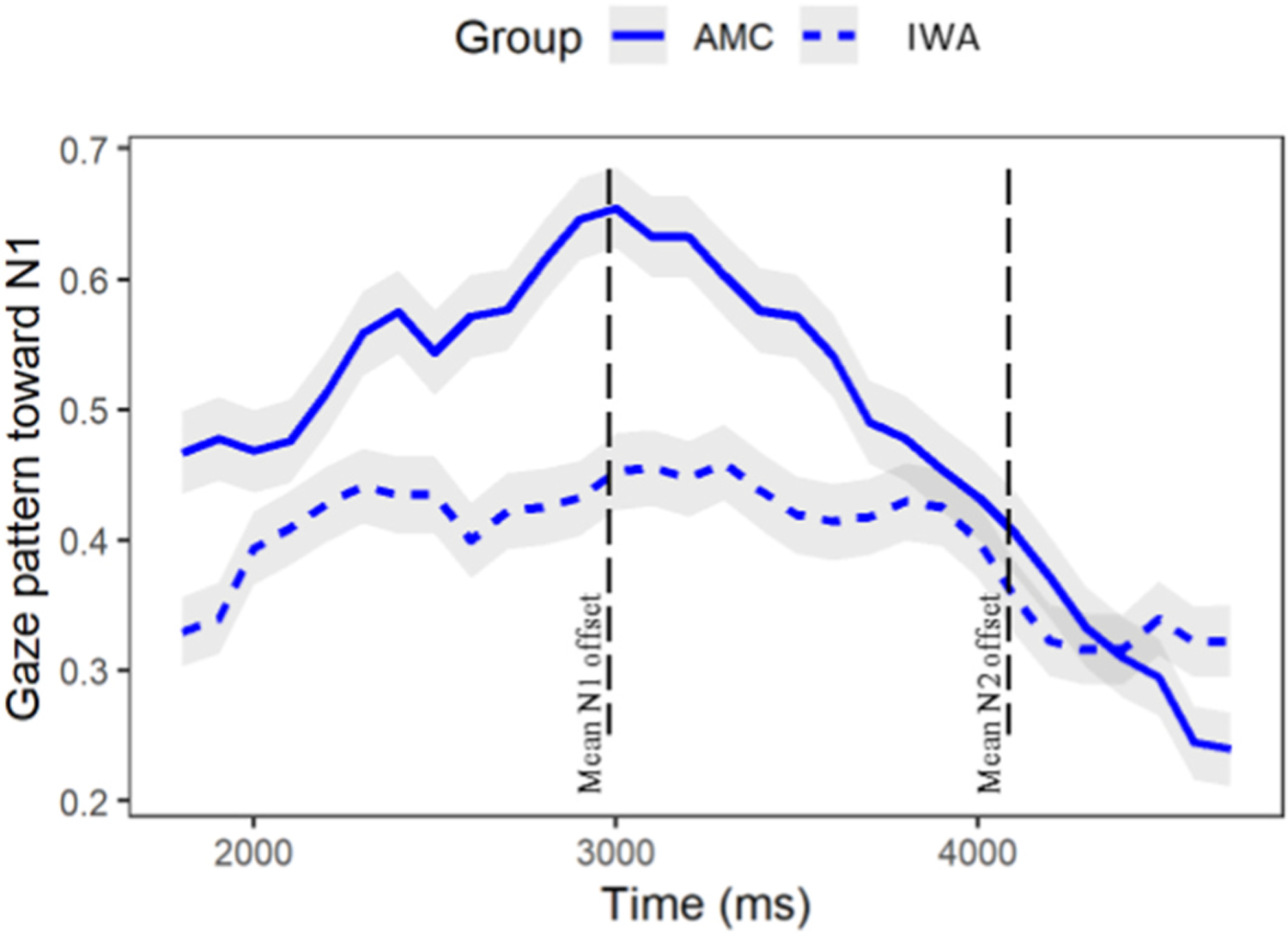
Comparing IWA with AMC on the timecourse of disengagement from N1 upon hearing N2 [… the model that noticed the dress …].

Having shown that both groups are sensitive to the lexical-semantic cue presented in the inanimate condition, we closely examined the time-course of sensitivity to it. Specifically, we asked whether IWA evince a temporal delay in their lexical processing as compared to AMCs. Within the same window of analysis that was examined for Research Question 1 (time window 1, see Table 2), we analyzed the activation and deactivation pattern of N1 by including a quadratic term in the model which can capture a curvilinear relationship between the proportion of looks over time for each group. To this end, we used growth curve analysis to see if there was a time-course difference between IWA and AMC in the rate of activation and consequently disengagement from N1 upon hearing the inanimate N2. Results (see Table 6) reveal a significant difference of group on the quadratic term (Quadratic: Estimate = 0.27, p < .05). As seen in Fig. 6, the IWA group had a shallower curvature of the gaze pattern toward N1 compared to AMC who had a steep rise and fall of gazes to N1. The positive coefficient in the interaction term revealed that the proportion of gazes toward N1 within the IWA group increases at a slower rate and as time progressed the proportion kept increasing and then eventually started to decrease. This is different from the AMC group as their proportions of gazes initially increased at a faster rate followed by a decreasing rate of looks to N1 (i.e., disengagement) upon hearing N2 (see Fig. 6).

### 3.5. Result of question 3 – downstream effect of the lexical-semantic cue on the dependency linking process

Recall that at the gap-site once the unaccusative verb is reached (e.g.,/fell/), successful dependency linking is evidenced as a reactivation of the N1. The gap-site window is specified to begin at the onset of the adverb that immediately precedes the verb (e.g.,/surprisingly/) until 2800ms afterward (time window 2, see Table 2). In this window of dependency linking, we inspected the presence of an interference effect by analyzing the gaze proportion of N1 (target or to-be-retrieved noun) versus N2 (intervening noun). Of importance is how mismatch in animacy feature is modulating the reactivation pattern of N1 and the interference effect from the intervening noun item in each group. To understand the downstream effect of the animacy manipulation at the gap site, for each group we built separate models and included the interaction of fixed effects of images (N1, N2) and condition. Of interest for the analysis here was the deactivation pattern of N2 as N1 was reactivated. We expected a steeper fall of N2 relative to N1 in the inanimate compared to the animate condition.

#### AMC group.

**Fig. 7.**
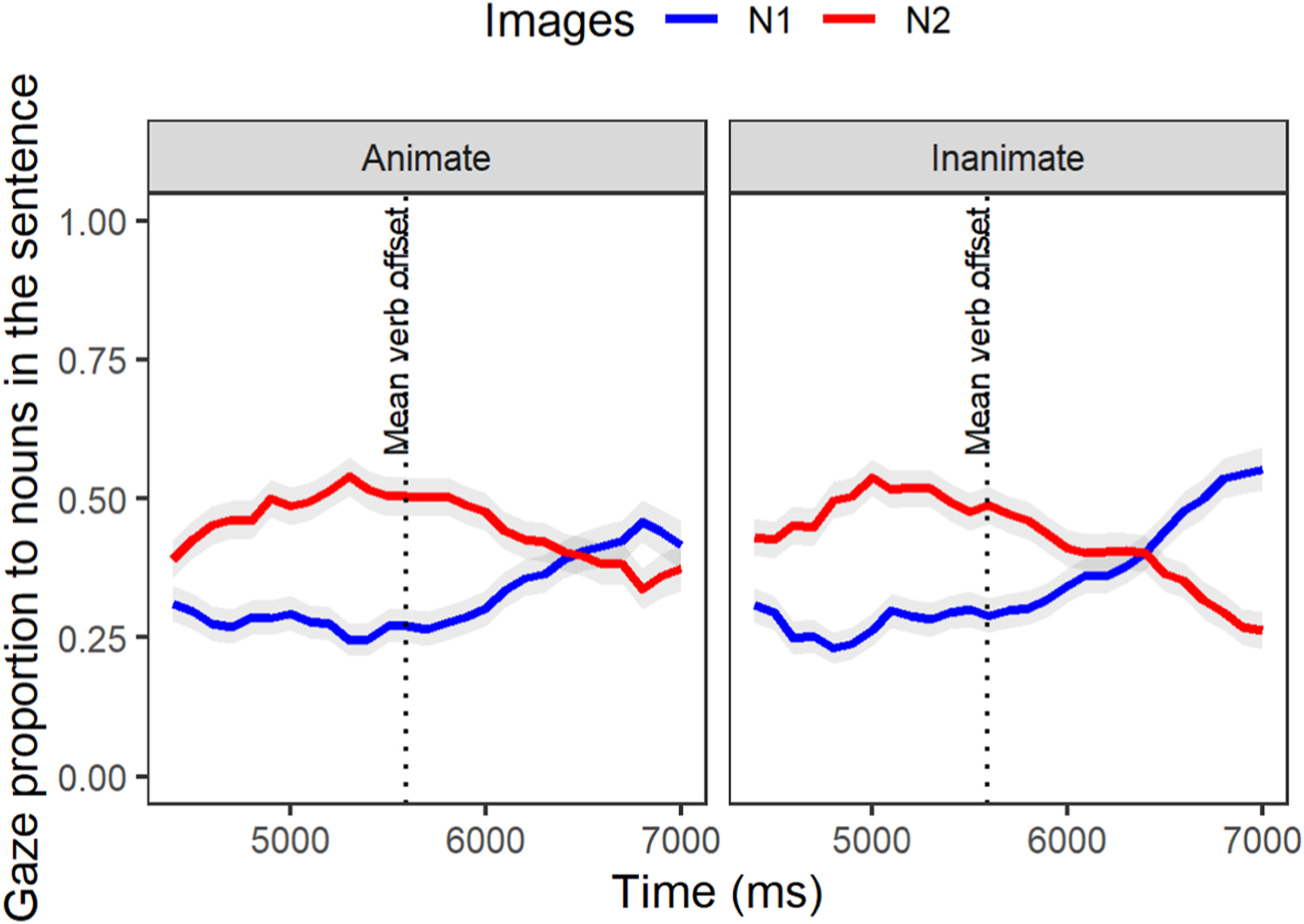
Gaze proportions of AMC individuals to the target (N1) and intervening (N2) items at the gap-site [… surprisingly fell during the evening gown …].

The individual parameter estimates for the AMC group (Fig. 7, Table 7) revealed a significant effect of condition on the difference between N1 and N2 at the linear level (Estimate = −0.4, p < .05). This suggested a faster rate of deactivation for N2 relative to N1 in the inanimate condition compared to the animate condition. This faster deactivation is evident in Fig. 7 in terms of steeper N2 deactivation and N1 activation slopes after the verb offset in the inanimate condition, as well as a greater difference in fixations to N1 versus N2 towards the end of the time window. This finding shows a clear influence of the pre-verbal lexical-semantic animacy cue on post-verbal dependency linking.

#### IWA group.

**Fig. 8.**
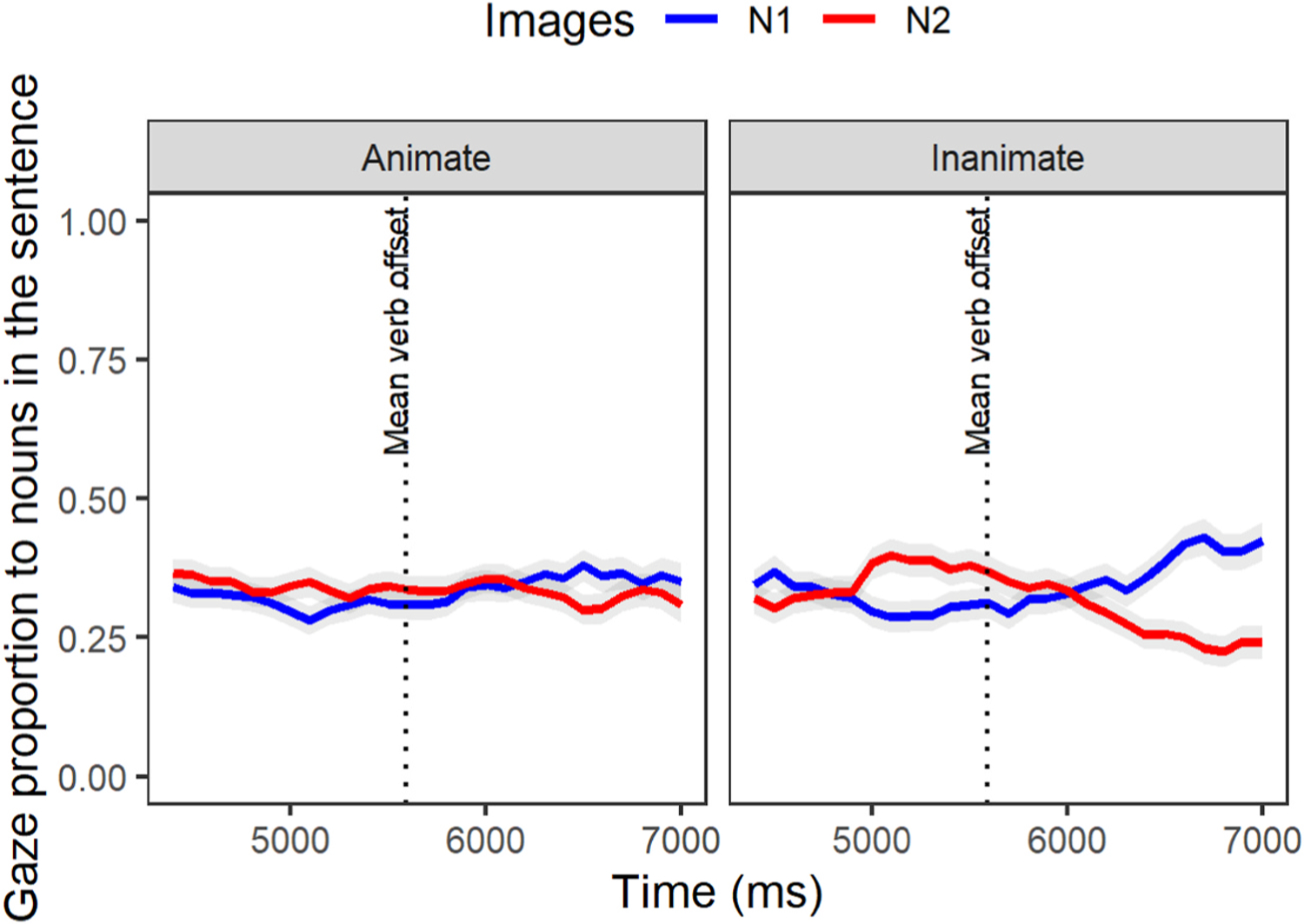
Gaze proportions of IWA individuals to the target (N1) and intervening (N2) items at the gap-site [… surprisingly fell during the evening gown …].

The individual parameter estimates for IWA (Fig. 8, Table 8) also revealed a significant effect of condition on the difference between N1 and N2 deactivation (Estimate = −0.3, p < .05). In IWA, while Fig. 8 reveals no clear differentiation between looks to N1 and N2 during post-verbal dependency linking, reflecting the expected similarity-based interference, the N1 is clearly reactivated while the N2 is deactivated in the inanimate condition. Thus overall, these results indicate a reduced interference effect of N2 in the inanimate condition for both groups.

## 4. General discussion

In the current study, we examined whether unimpaired listeners and individuals with aphasia (IWA) can use lexical-semantic cuing in real-time during comprehension of unaccusative verbs that require long-distance dependency linking within canonically-structured sentences. First, we examined the sensitivity of listeners in both groups to a lexical-sematic animacy cue. Second, we examined the time course of processing the lexical-semantic cue and its effects on N1 activation and deactivation in individuals with aphasia compared to the age-matched unimpaired control (AMC) individuals. Third, we assessed whether the lexical-semantic cue has a downstream effect on dependency linking at the gap-site for both groups. We used animacy as the semantic cue to reduce the similarity-based interference effect. We employed an eye-tracking visual world paradigm to capture the real-time processing stream during auditory comprehension. We confirmed our predicted hypothesis that listeners in both groups would be sensitive to the semantic-lexical cue in the inanimate condition. We also confirmed our predicted hypothesis for individuals with aphasia that they would show sensitivity to the lexical-semantic cue, but with a delay in processing. Finally, we confirmed in both groups that the lexical-semantic cue resulted in significant reduction in a similarity-based interference effect throughout the sentence processing stream, with markedly changed gaze patterns during dependency linking at the gap-site.

### 4.1. Sensitivities to lexical-semantic cues across the sentence processing timeline (research questions 1 and 3)

Our results from both AMC and IWA confirmed the predictions of the similarity-based interference approach. We found that IWA were experiencing an interference effect during real-time dependency linking and integration. Specifically, in windows where the argument verbs had to be specified (time window 1 and 2, see Table 2), IWA showed a lack of ability in distinguishing each noun phrase (See Figs. 6 and 8). However, when the level of competition was reduced (via a semantic-animacy manipulation), IWA showed a facilitation effect, as they were able to distinguish between the two noun phrases and reactivate the antecedent (N1) at the gap-site. These results revealed that IWA were able to access the lexical-semantic representation of the words and used it during their auditory processing in real-time. However, vulnerability in the processing system of IWA was apparent when there was a potential case of interference in the animate condition. This is in line with predictions of the Intervener hypothesis (Engel et al., 2018; Sheppard et al., 2015), which emphasizes that computing the dependency relationship for IWA becomes more difficult when there is a structurally similar intervener that can pose competition for the to-be-retrieved target item during the dependency linking process.

### 4.2. Time course of sensitivity to the lexical-semantic cues (research question 2)

Individuals with aphasia (IWA) were shown to be sensitive to the animacy information, even though they appeared to process it slower than AMC participants. This was apparent in delayed deactivation of N1 in IWA when N2 was encountered. The delay in processing of IWA has also been shown in other studies and using different methodologies. For instance, in a visual-world eyetracking study, Mack and colleagues examined whether IWA could utilize verb meaning to predict and enhance the integration of a following noun argument (Mack et al., 2013). Nine agrammatic aphasic adults and ten age-matched controls took part in the two experiments. During Experiment 1, the participants’ eye movements were tracked while looking at an array of four objects (such as a jar, plate, stick, and pencil) as they were listening to sentences that included either a restrictive verb that was semantically compatible only with the target object or an unrestrictive verb that was compatible with all four objects (e.g., “Susan will open/break the jar”). The results revealed that IWA showed a lower proportion of gazes toward the target object as compared to the control participants within the first 500ms following the verb offset, which corresponded with the presentation of the target noun phrase. This suggests that the IWA had delayed access to the lexical representations of nouns (for similar findings, see Choy, 2011; Love et al., 2008; Prather et al., 1992; 1997).

Mack et al.’s second experiment replicated this finding in the context of incomplete sentences (e.g., Susan will open/break the …). The impact of verb type on IWA was significantly delayed compared to the control participants. Overall, these findings provide evidence that IWA have a lexical processing delay that explains their sentence processing deficits. In another study using a self-paced listening task, DeDe (2012) found that individuals with anomia have delayed processing of lexical-semantic and prosodic information (DeDe, 2012). Moreover, in a study using Event-related potentials (ERP), Sheppard and colleagues found that IWA (individuals with agrammatic aphasia) showed a delayed N400 effect (Sheppard et al., 2017). In this ERP study, the individuals with agrammatic aphasia were unable to integrate semantic and prosodic cues to predict upcoming syntactic structure and prevent garden path effects in sentences with incongruent prosody.

These findings described above converge with the current findings of overall lower offline comprehension accuracy in IWA, particularly in the animate condition; further, they confirm that a delay in lexical processing, including lexical integration challenges, can negatively impact sentence processing success (Choy & Thompson, 2010; Dickey et al., 2007; Love et al., 2008; Thompson & Choy, 2009). The delayed lexical processing can hinder the ability of IWA to integrate relevant information while processing the sentence, leading to difficulties in comprehending the relationship between sentence constituents. Additionally, processing complex sentence structures involving embedded clauses or long-distance dependencies that require timely gap-filling mechanisms can be challenging for IWA. The nature of this delay in processing lexical-semantic representations can have a multifactorial etiology. Jefferies and Lambon Ralph (Jefferies & Lambon Ralph, 2006) have proposed that deficits in processing can reflect impairments of “semantic control processes”. In other words, impairment in processes that allow relevant aspects of knowledge to be attended to while irrelevant information is inhibited (Jefferies, 2013; Jefferies et al., 2008; Jefferies & Lambon Ralph, 2006; Mirman & Britt, 2014). In case of left-hemisphere stroke, damage to networks involving inferior prefrontal cortex, posterior middle temporal gyrus and the intraparietal sulcus (Noonan et al., 2013; Rodd et al., 2005; Thompson-Schill et al., 1997) is likely to results in an impaired ability to retrieve weak or less automatic semantic associations, to resolve competition between multiple competing representations, and to inhibit irrelevant semantic information (Paul Hoffman et al., 2011; Jefferies & Lambon Ralph, 2006; Noonan et al., 2010). For a full discussion of these control processes, see (Hoffman, 2018; Hoffman et al., 2009; Hoffman et al., 2013; Hoffman et al., 2011).

The current study adds to these previous findings (e.g., Caramazza & Zurif, 1976) by providing an online processing account of how animacy mismatch can improve comprehension of sentences in IWA. Specifically, our study used eye-tracking technology to demonstrate that an animacy mismatch between nouns can facilitate online sentence processing in IWA, even when the animacy mismatch is not necessary for correct sentence comprehension. This nuance adds to the previous account and has important clinical implications for understanding and improving language processing in IWA.

### 4.3. Limitations and implications

There are limitations to the current study that can be addressed in future research. First, there were a relatively small number of participants within the aphasia group. This is particularly important because inter-participant variability is inherent in individuals with aphasia, thus it is essential that the questions in the current study be addressed with a larger number of participants in future studies. Second, while GCA is widely used in the analysis of eye-tracking data (e.g., Mirman et al., 2008; Mirman et al., 2011), there are some limitations to this method that require careful assessment (Huang & Snedeker, 2020). One issue is inherent to eye-tracking: including high autocorrelation in the data. To reduce the autocorrelation of gaze signals, we binned the data by averaging it every 100ms. We also used linear-mixed effect modeling and accounted for the random effects of individuals and stimulus items on each of the orthogonal time terms included in the model. This analytic approach can help reduce autocorrelation by capturing variation in the data that is not explained by fixed effects and can thus reduce unobserved heterogeneity in the data. Another issue with GCA is the lack of clear guidelines for determining which temporal parameters and interactions to include in the model. It is important to note that including quadratic, cubic, and quartic parameters without clear cognitive interpretations increases the risk of false positives (see Huang & Snedeker, 2020 for references). To mitigate this risk, we pre-specified analyses with well-motivated linking hypotheses and only included the slop and quadratic effects in our models. We acknowledge that alternative methods have been developed to address the issues with modeling visual world paradigm data, as demonstrated by studies such as Cho et al. (2018) and Oleson, Cavanaugh, McMurray, and Brown (2017). Third, our study did not specifically investigate the nature of the underlying memory and cognitive control system through experimental manipulation or inclusion of cognitive measures. Such memory and cognitive processes may support individual differences in sensitivity to the semantic cue and in the response timecourse during sentence comprehension. Finally, our study did not specifically examine which lesion characteristics impacted the extent of benefiting from the lexical-semantic cueing in real-time. We believe that the current study makes an important initial contribution in suggesting that a semantic cue at the sentence level, specifically an animacy-mismatch between a target and intervening noun, can considerably and reliably support sentence processing in an initial group of individuals with aphasia. It should be noted that there may be other ways to define similarity between sentence constituents beyond animacy (e.g., a match in grammatical gender or another shared semantic category) and that this is a promising avenue for future research. The findings from the current study will provide a useful basis for new research and has clear implications for intervention in IWA. Specifically, in interventions targeting complex sentence processing in IWA, an initial step to increase comprehensibility of a sentence may be an animacy mismatch between nouns as was undertaken here. Instead, if a speech-language pathologist desires that their client with aphasia focus solely on syntactic processes during an intervention session, an animacy match between noun constituents may be a valuable strategy. With such an animacy match, individuals may no longer be able to rely on their semantic heuristic knowledge to process a sentence. In such cases, they would need to focus on the syntactic structure of the sentence, which can be a valuable strategy in interventions targeting syntactic processing.

## 5. Conclusions

The current study provides insight on how lexical-semantic representations are encoded and processed during sentence comprehension in real-time by bringing evidence from both neurologically unimpaired as well as impaired listeners. Our results revealed that reduction in representational interference via a lexical-semantic manipulation can facilitate real-time sentence processing of unimpaired populations and those with aphasia. Yet, individuals with aphasia revealed a temporal delay in their sensitivity to lexical-semantic manipulation during real-time processing.

## Figures and Tables

**Table 1:** Characteristics of IWA (n = 11) and neurotypical age-matched control (AMC) participants (n = 11).

IWA	Sex	Years Post-Stroke	Age at Testing	Years of Education	Aphasia Subtype	Lesion Location	BDAE-v3	WAB-R AQ	SOAP-SR (%)	SOAP- OR (%)
**S1**	M	15	55	17	Mixed non-fluent	L lesion, IFG (BA 44/BA45) w/posterior ext.	4	67.7	60	40
**S2**	M	18	66	15	Anomic	L anterior cerebral and middle cerebral infarct	4	95.4	100	90
**S3**	M	9	67	20	Broca	L lesion posterior IFG (BA 44) w/posterior ext.	2	82.6	100	30
**S4**	M	8	63	16	Broca /Anomic	L IPL w/posterior ext. sparing STG	4	90.5	75	55
**S5**	F	16	42	NA	NA	L MCA infarct	2	75.7	80	30
**S6**	F	7	65	16	Anomic	L MCA infarct w/subcortical ext.	4	95.8	100	100
**S7**	F	6	64	16	Broca	L MCA infarct	3	92.4	100	70
**S8**	F	4	64	12	Broca	L MCA infarct	3	NA	80	60
**S9**	M	4	59	12	Broca	L MCA infarct	2	28.2	80	40
**S10**	F	6	76	12	Broca	L superior temporal lesion	3	88.2	90	40
**S11**	M	1	57	16	Broca	L MCA infarct	4.5	98.4	100	60
**AMC Group**	Ages 57–66 years (mean = ~61.9); 7 females, 4 males; Education 14–18 years (mean = 15.7)									

L = left; BA = Brodmann area; IPL = inferior parietal lobule; STG = superior temporal gyrus; MCA = middle cerebral artery; BDAE = Boston Diagnostic Aphasia Examination (0 = no useable speech or auditory comprehension, 5 = minimal discernible speech handicap); SOAP SR = Average percent correct on subject relative items from the SOAP Test of Auditory Sentence Comprehension; SOAP OR = Average percent correct of object relative items from the SOAP Test of Auditory Sentence Comprehension; NA = Data is not available.

**Table 2:** The pre-identified windows of interest across the sentences.

	Time window 1 Embedded clause	Time window 2 Dependency processing
**Mean onset time**	1800–4800	4400–7000

**Table 3:** The mean proportion accuracy of comprehension questions across groups and conditions.

	Animate	Inanimate
AMC (n = 11)	0.94 (0.28)	0.98 (0.14)
IWA (n = 11)	0.69 (0.46)	0.75 (0.43)

**Table 4:** Results of GCA analysis for time window 1 (interference in embedded clause) within the AMC group.

Predictors	Estimates	CI	p
Intercept	0.38	0.34 – 0.42	<0.001
Linear	−0.20	−0.38 – −0.02	0.031
Image[N2]	−0.08	−0.13 – −0.02	0.004
Condition[Inanimate]	0.10	0.06 – 0.13	<0.001
Linear*Image[N2]	0.45	0.21–0.69	<0.001
Linear*Condition[Inanimate]	−0.11	−0.24 – 0.02	0.095
Image[N2]*Condition[Inanimate]	−0.16	−0.18 – −0.14	**<0.001**
Linear*Image[N2]*Condition[Inanimate]	0.25	0.13 – 0.37	**<0.001**

Note: The table shows the summary results of the model for the AMC group, which included the interaction between images and conditions on the intercept and linear time terms. Results in **boldface** are presented in the text.

**Table 5:** Results of GCA analysis for time window 1 (interference in embedded clause) within the IWA group.

Predictors	Estimates	CI	p
Intercept	0.34	0.30 – 0.38	<0.001
Linear	−0.00	−0.14 – 0.14	0.998
Image[N2]	−0.04	−0.09 – 0.00	0.062
Condition[Inanimate]	0.05	0.02 – 0.08	<0.001
Linear*Image[N2]	0.18	0.01 – 0.35	0.040
Linear*Condition[Inanimate]	−0.13	−0.24 – −0.02	0.025
Image[N2]*Condition[Inanimate]	−0.12	−0.14 – −0.10	**<0.001**
Linear*Image[N2]*Condition[Inanimate]	0.22	0.11 – 0.32	**<0.001**

Note: The table shows the summary results of the model for the IWA group, which included the interaction between images and conditions on the intercept and linear time terms. Results in **boldface** are presented in the text.

**Table 6:** Results of GCA analysis for time window 1. Group differences in the time-course of sensitivity to the cue.

Predictors	Estimates	CI	p
Intercept	0.49	0.42 – 0.56	<0.001
Linear	−0.37	−0.64 – −0.11	0.006
Quadratic	−0.49	−0.69 – −0.28	<0.001
Group[IWA]	−0.09	−0.17 – −0.01	**0.022**
Linear*Group[IWA]	0.24	−0.06 – 0.55	0.110
Quadratic*Group[IWA]	0.27	0.03 – 0.50	**0.026**

Note: The table shows the result of the model including the effect of group on the intercept, linear and quadratic time terms. Results in **boldface** are presented in the text.

**Table 7:** Results of GCA analysis for time window 2 (dependency processing) within the AMC group.

Predictors	Estimates	CI	p
Intercept	0.33	0.28 – 0.39	<0.001
Linear	0.23	0.04 – 0.43	0.018
Quadratic	0.13	0.01– 0.26	0.038
Image[N2]	0.10	0.02–0.17	0.017
Condition[Inanimate]	0.02	−0.01 – 0.04	0.274
Linear*Image[N2]	−0.33	−0.61 – −0.06	0.016
Quadratic*Image[N2]	−0.32	−0.50 – −0.15	<0.001
Linear*Condition[Inanimate]	0.20	0.10 – 0.31	<0.001
Quadratic*Condition[Inanimate]	0.07	−0.03 – 0.17	0.187
Image[N2]*Condition[Inanimate]	−0.04	−0.07 – −0.02	0.001
Linear*Image[N2]*Condition[Inanimate]	−0.37	−0.51 – −0.24	**<0.001**
Quadratic*Image[N2]*Condition[Inanimate]	−0.06	−0.20 – 0.07	0.347

Note: The table shows the summary results of the model for the AMC group, which included the interaction between images and conditions on the intercept, linear, and quadratic time terms. Results in **boldface** are presented in the text.

**Table 8:** Results of GCA analysis for time window 2 (dependency processing) for the IWA group.

Predictors	Estimates	CI	p
Intercept	0.33	0.28 – 0.38	<0.001
Linear	0.08	−0.07 – 0.23	0.279
Quadratic	0.05	−0.04 – 0.14	0.241
Image[N2]	0.01	−0.05 – 0.08	0.693
Condition[Inanimate]	0.01	−0.02 – 0.04	0.570
Linear*Image[N2]	−0.12	−0.33 – 0.08	0.241
Quadratic*Image[N2]	−0.05	−0.17 – 0.08	0.475
Linear*Condition[Inanimate]	0.10	−0.00 – 0.20	0.060
Quadratic*Condition[Inanimate]	0.10	0.02 – 0.19	0.015
Image[N2]*Condition[Inanimate]	−0.04	−0.06 – −0.02	<0.001
Linear*Image[N2]*Condition[Inanimate]	−0.27	−0.39 – −0.16	**<0.001**
Quadratic*Image[N2]*Condition[Inanimate]	−0.28	−0.39 – −0.17	<0.001

Note: The table shows the summary results of the model for the IWA group, which included the interaction between images and conditions on the intercept, linear, and quadratic time terms. Results in **boldface** are presented in the text.

## Data Availability

Data will be made available on request.

## Footnotes

1 In this manuscript we also use [N] as an abbreviation for noun. In this study, the first encountered noun in the sentence is referred to as N1 and the second noun as N2.

2 Structurally, proper nouns like “Christopher” are standalone words and do not typically take any modifiers, while definite noun phrases like “the general” consist of a determiner followed by a noun and can be modified by adjectives or other words. Semantically, the difference between a proper noun (Christopher) and a definite noun phrase (the general) is that the former refers to a specific, unique individual, while the latter refers to a particular entity within a group or class.

3 **Table:**

*Who* subject-extracted	Who__pushed the fireman yesterday afternoon?
*Who* object-extracted	Who did the fireman push __ yesterday afternoon?
*Which* subject-extracted	Which mailman __ pushed the fireman yesterday afternoon?
*Which* object-extracted ______	Which mailman did the fireman push __ yesterday afternoon?

4 Active: The tiger chased the lion.Passive: The lion was chased by the tiger.

5 Double-Object: The sister mailed the brother the letter.Prepositional-phrase Object: The sister mailed the letter to the brother.

6 Unaccusative sentences can be structured in both canonical and non-canonical forms. In the canonical form, the sentence may have a single subject followed by an intransitive verb (e.g., “The vase broke”). In the non-canonical form, the sentence may have a prepositional phrase followed by the intransitive verb, which is followed by the subject (e.g., “On the table sat the vase”).

7 (1) There disappeared three girls after class.(2) *The principal disappeared three girls after class.(3) *Three girls were disappeared after class (by the principal).

8 For our first research question, our main interest was to examine the linear pattern capturing the rate of gaze toward N1 and N2, rather than any potential curvilinear patterns. Additionally, we determined that including a quadratic term would not improve the model fit or increase the accuracy of the estimates. It is worth noting that adding a quadratic term can increase the complexity of the model and the risk of overfitting, which is especially important to avoid when it is not justified based on the research question (Huang & Snedeker, 2020).

## Appendix. Stimuli employed in the current study, including the initial scene-setting prepositional phrase (PP), the core discourse of interest in each trial, and comprehension questions that followed trials together with their correct responses

**Table:**

#	Animacy	PP	Discourse of Interest	Comprehension Questions	Response
1	animate	Yesterday at the music festival,	The guitarist that noticed the drummer frantically arrived before the performance.	Did the guitarist arrive before the performance?	Y
2	inanimate	Yesterday at the music festival,	The guitarist that noticed the drumset frantically arrived before the performance.	Did the guitarist arrive before the performance?	Y
3	animate	Last night at the Halloween party,	The pirate that observed the wizard suddenly emerged before the costume contest.	Did the wizard emerge before the costume contest?	N
4	inanimate	Last night at the Halloween party,	The pirate that observed the pumpkin suddenly emerged before the costume contest.	Did the pirate emerge before the costume contest?	Y
5	animate	Last weekend at the dinner party,	The waitress that saw the bartender abruptly vanished before the appetizer course.	Did the waitress vanish before the appetizer course?	Y
6	inanimate	Last weekend at the dinner party,	The waitress that saw the napkin abruptly vanished before the appetizer course.	Did the napkin vanish before the appetizer course?	N
7	animate	Last week at the street fair,	The jeweler that saw the grocer immediately languished after the flood of customers.	Did grocer languish after the flood of customers?	N
8	inanimate	Last week at the street fair,	The jeweler that saw the foodtruck immediately languished after the flood of customers.	Did jeweler languish after the flood of customers?	Y
9	animate	This morning at the circus,	The acrobat that observed the clown noisily remained during the trapeze act.	Did the acrobat remain during the trapeze act?	Y
10	inanimate	This morning at the circus,	The acrobat that observed the balloon noisily remained during the trapeze act.	Did the balloon remain during the trapeze act?	N
11	animate	A few nights ago at the tropical plant exposition,	The gardener that saw the florist loudly awoke in the middle of the sales pitch.	Did the gardener awake in the middle of the sales pitch?	Y
12	inanimate	A few nights ago at the tropical plant exposition,	The gardener that saw the flower loudly awoke in the middle of the sales pitch.	Did the gardener awake in the middle of the sales pitch?	Y
13	animate	Yesterday afternoon at the baseball game,	The pitcher that noticed the umpire quietly arose before the opening ceremony.	Did the pitcher arise before the opening ceremony?	Y
14	inanimate	Yesterday afternoon at the baseball game,	The pitcher that noticed the peanuts quietly arose before the opening ceremony.	Did the pitcher arise before the opening ceremony?	Y
15	animate	Last month at the triathlon,	The cyclist that saw the sprinter silently flourished during the final event.	Did the sprinter flourish during the final event?	N
16	inanimate	Last month at the triathlon,	The cyclist that saw the waterbottle silently flourished during the final event.	Did the cyclist flourish during the final event?	Y
17	animate	A few days ago at the soccer match.	The referee that saw the goalie quickly departed after the second half.	Did the referee depart after the second half?	Y
18	inanimate	A few days ago at the soccer match,	The referee that saw the whistle quickly departed after the second half.	Did the referee depart after the second half?	Y
19	animate	This afternoon at the extreme sports competition,	The skateboarder that saw the skydiver unexpectedly disappeared after the awards ceremony.	Did the skateboarder disappear after the awards ceremony?	Y
20	inanimate	This afternoon at the extreme sports competition,	The skateboarder that saw the goggles unexpectedly disappeared after the awards ceremony.	Did the goggles disappear after the award ceremony?	N
21	animate	This evening at the fashion show,	The model that noticed the designer surprisingly fell during the evening gown showcase.	Did the model fall during the evening gown showcase?	Y
22	inanimate	This evening at the fashion show,	The model that noticed the dress surprisingly fell during the evening gown showcase.	Did the dress fall during the evening gown showcase?	N
23	animate	A few weeks ago at the trailhead,	The hiker that observed the guide thankfully lived after the unexpected rock slide.	Did the guide live after the unexpected rock slide?	N
24	inanimate	A few weeks ago at the trailhead,	The hiker that observed the map thankfully lived after the unexpected rock slide.	Did the hiker live after the unexpected rock slide?	Y
25	animate	Last Monday at the emergency evacuation.	The police officer that noticed the paramedic carelessly stood during the chaos.	Did the police officer stand during the chaos?	Y
26	inanimate	Last Monday at the emergency evacuation,	The police officer that noticed the helicopter carelessly stood during the chaos.	Did the police officer stand during the chaos?	Y
27	animate	Three months ago at the hotel grand opening,	The bellhop that saw the receptionist finally appeared during the ribbon cutting event.	Did the receptionist appear during the ribbon cutting event?	N
28	inanimate	Three months ago at the hotel grand opening,	The bellhop that saw the chandelier finally appeared during the ribbon cutting event.	Did the chandelier appear during the ribbon cutting event?	N
29	animate	A few months ago at the county fair,	The performer that noticed the fortune teller excitedly soared during the rollercoaster ride.	Did the performer soar during the rollercoaster ride?	Y
30	inanimate	A few months ago at the county fair,	The performer that noticed the fire cracker excitedly soared during the rollercoaster ride.	Did the performer soar during the rollercoaster ride?	Y
31	animate	Yesterday at the sporting goods store,	The tennis player that observed the fisherman frantically arrived in the middle of the clearance sale.	Did the fisherman arrive in the middle of the clearance sale?	N
32	inanimate	Yesterday at the sporting goods store,	The tennis player that observed the fishing pole frantically arrived in the middle of the clearance sale.	Did the tennis player arrive in the middle of the clearance sale?	Y
33	animate	Last night at the basketball game,	The point guard that noticed the coach suddenly emerged before the last quarter.	Did the point guard emerge before the last quarter?	Y
34	inanimate	Last night at the basketball game,	The point guard that noticed the clock suddenly emerged before the last quarter.	Did the point guard emerge before the last quarter?	Y
35	animate	Last weekend at the Broadway play,	The actress that observed the producer abruptly vanished before the brief intermission.	Did the actress vanish before the brief intermission?	Y
36	inanimate	Last weekend at the Broadway play,	The actress that observed the piano abruptly vanished before the brief intermission.	Did the piano vanish before the brief intermission?	N
37	animate	Last week at the symphony,	The conductor that observed the violinist immediately languished after several concerts.	Did the conductor languish after several concerts?	Y
38	inanimate	Last week at the symphony,	The conductor that observed the ticket immediately languished after several concerts.	Did the ticket languish after several concerts?	N
39	animate	This morning at the international travel seminar,	The flight attendant that saw the speaker noisily remained during the safety demonstration.	Did the flight attendant remain during the safety demonstration?	Y
40	inanimate	This morning at the international travel seminar,	The flight attendant that saw the laptop noisily remained during the safety demonstration.	Did the laptop remain during the safety demonstration?	N
41	animate	A few nights ago at the movie theatre,	The girl that observed the boy loudly awoke during the horror film.	Did the girl awake during the horror film?	Y
42	inanimate	A few nights ago at the movie theatre,	The girl that observed the popcorn loudly awoke during the horror film.	Did the girl awake during the horror film?	Y
43	animate	Yesterday afternoon at the beach,	The surfer that noticed the scuba diver quietly arose before high tide.	Did the scuba diver arise before high tide?	N
44	inanimate	Yesterday afternoon at the beach,	The surfer that noticed the sunglasses quietly arose before high tide.	Did the surfer arise before high tide?	Y
45	animate	Last year at the winter Olympics,	The ice skater that noticed the snow boarder silently flourished during the final round.	Did the ice skater flourish during the final round?	Y
46	inanimate	Last year at the winter Olympics,	The ice skater that noticed the ski jacket silently flourished during the final round.	Did the ice skater flourish during the final round?	Y
47	animate	A few days ago at the book release party,	The author that observed the illustrator quickly departed after the opening remarks.	Did the illustrator depart after the opening remarks?	N
48	inanimate	A few days ago at the book release party,	The author that observed the microphone quickly departed after the opening remarks.	Did the author depart after the opening remarks?	Y
49	animate	This afternoon at the dance recital,	The ballerina that saw the showgirl unexpectedly disappeared in the middle of the routine.	Did the ballerina disappear in the middle of the routine?	Y
50	inanimate	This afternoon at the dance recital,	The ballerina that saw the stage door unexpectedly disappeared in the middle of the routine.	Did the stage door disappear in the middle of the routine?	N
51	animate	This evening at the ice-skating rink,	The zamboni driver that observed the hockey player surprisingly fell after the open skate session.	Did the hockey player fall after the open skate session?	N
52	inanimate	This evening at the ice-skating rink,	The zamboni driver that observed the locker surprisingly fell after the open skate session.	Did the locker fall after the open skate session?	N
53	animate	A few weeks ago at the country western festival	The cowgirl that noticed the rodeo clown thankfully lived after the bull stampede.	Did the cowgirl live after the bull stampede?	Y
54	inanimate	A few weeks ago at the country western festival	The cowgirl that noticed the barrel thankfully lived after the bull stampede.	Did the cowgirl live after the bull stampede?	Y
55	animate	Last Monday at the cooking demonstration,	The chef that noticed the baker carelessly stood after the sudden explosion.	Did the baker stand after the sudden explosion?	N
56	inanimate	Last Monday at the cooking demonstration,	The chef that noticed the bowl carelessly stood after the sudden explosion.	Did the chef stand after the sudden explosion?	Y
57	animate	Three months ago at the wedding reception,	The bride that observed the groom finally appeared in the middle of the champagne toast.	Did the bride appear in the middle of the champagne toast?	Y
58	inanimate	Three months ago at the wedding reception,	The bride that observed the gift finally appeared in the middle of the champagne toast.	Did the gift appear in the middle of the champagne toast?	N
59	animate	A few months ago at the amusment park,	The vendor that saw the character excitedly soared during the bungee jump experience.	Did the vendor soar during the bungee jump experience?	Y
60	inanimate	A few months ago at the amusment park,	The vendor that saw the cotton candy excitedly soared during the bungee jump experience.	Did the vendor soar during the bungee jump experience?	Y
